# Trachoma and Yaws: Common Ground?

**DOI:** 10.1371/journal.pntd.0004071

**Published:** 2015-12-03

**Authors:** Anthony W. Solomon, Michael Marks, Diana L. Martin, Alexei Mikhailov, Rebecca M. Flueckiger, Oriol Mitjà, Kingsley Asiedu, Jean Jannin, Dirk Engels, David C. W. Mabey

**Affiliations:** 1 World Health Organization, Department of Control of Neglected Tropical Diseases, Geneva, Switzerland; 2 Clinical Research Department, Faculty of Infectious and Tropical Diseases, London School of Hygiene & Tropical Medicine, London, United Kingdom; 3 The Hospital for Tropical Diseases, London, United Kingdom; 4 Division of Parasitic Diseases and Malaria, Centers for Disease Control and Prevention, Atlanta, Georgia, United States of America; 5 International Trachoma Initiative, Task Force for Global Health, Atlanta, Georgia, United States of America; 6 Barcelona Institute for Global Health, Barcelona Centre for International Health Research, Hospital Clinic, University of Barcelona, Barcelona, Spain; 7 Lihir Medical Centre—International SOS, Newcrest Mining, Lihir Island, Papua New Guinea; University of California San Diego School of Medicine, UNITED STATES

Trachoma is an important cause of blindness. The causative organism is an intracellular bacterium, *Chlamydia trachomatis*, which is susceptible to single-dose azithromycin [1]. A World Health Organization (WHO)-led program aims to eliminate trachoma as a public health problem globally by 2020 [2]. Yaws is a cause of skin, bone, and cartilage disease. The causative organism is a spirochaete bacterium, *Treponema pallidum* ssp. *pertenue*, which is susceptible to single-dose azithromycin [3]. A WHO-led program aims to eradicate yaws globally by 2020 [4].

These diseases are both found in hard-to-reach populations—they affect the poorest people living in the most remote areas of the countries where they’re found—and have some apparent similarity in the methods recommended to counter them. Maximum synergy between programs is possible only if the two diseases affect the same communities, and if program goals permit alignment of work. Trachoma’s elimination as a public health problem means “the reduction of disease incidence, prevalence, morbidity or mortality to a locally acceptable level as a result of deliberate efforts” [5], whereas yaws eradication requires “permanent reduction to zero of the worldwide incidence of infection caused by a specific agent as a result of deliberate efforts” [5]—a quite different goal. This symposium reviews the extent to which the epidemiologies of and management strategies for these diseases actually overlap, to determine areas for mutually beneficial collaboration.

## Has Global Control of Either Yaws or Trachoma Been Attempted Previously?

In 1949, the Haiti yaws control program began to employ long-acting penicillin injections, supplanting earlier efforts using toxic and poorly efficacious arsenicals. Organized with military-style intensity, the campaign became a resounding success, prompting the first global commitment to eradicate yaws [6,7]. Over the next several decades, the incidence of yaws declined to such a level that it was believed to be disappearing altogether; as a consequence, mobile teams were widely disbanded or repurposed. Recrudescence followed [8]. A second, generally half-hearted effort to eradicate yaws was initiated in the late 1970s, which was again unsuccessful. In the 1990s, national elimination programs interrupted transmission in Ecuador and India [6]. Then, in 2010–2011, a randomised trial conducted in Papua New Guinea demonstrated that a single oral dose of azithromycin is at least as efficacious as intramuscular penicillin in achieving cure [3], stimulating development of a WHO-endorsed strategy [9] for a third attempt at global yaws eradication.

In the 1950s, several countries launched campaigns attempting to control trachoma using tetracycline eye ointment [7]. From the recipients’ perspective, ocular tetracycline is not an ideal therapy: it is difficult to apply, irritant to the conjunctivae, and, for refractive reasons, blurs vision for a few minutes after application. Prolonged use—six weeks continuous or six months intermittent treatment—is recommended for cure. Because most individuals provided with tetracycline ointment are asymptomatic, it is not surprising that results were mixed. It was soon realised that such campaigns would be resource-intensive and lengthy, yet might not work. Trachoma, it was decided, was not a suitable disease for control via mass treatment [7]. The 1990s discovery that single-dose azithromycin was at least as effective as a six-week course of tetracycline eye ointment [10] reignited enthusiasm and led to development of the community-based “SAFE” strategy for global elimination, in which mass treatment with antibiotics (A) is combined with facial cleanliness (F) and environmental improvement (E) to reduce transmission, with surgery (S) being provided for advanced disease.

## How Is the Need for Interventions against These Infections Determined?

For trachoma, population-based prevalence survey data are important, because the district-level prevalence in 1- to 9-year-old children of the sign “trachomatous inflammation—follicular” (TF) [11] is the key index for determining whether or not to implement the A, F, and E components of SAFE [12]. However, after several years of SAFE implementation, the positive predictive value of TF for ocular *C*. *trachomatis* infection often decreases, at both the individual and community level. Determining when to stop antibiotic mass distribution may therefore be problematic; research to address this is underway. For yaws, baseline prevalence estimates are not necessarily needed because the WHO Morges strategy for eradication is required in any population in which at least one clinically suspicious case is confirmed serologically to be yaws. WHO has produced a picture guide [13] to aid clinical diagnosis; a point-of-care antibody test performs well [14,15], and efforts are in progress to make this test available to programs. Intervention (the WHO Morges strategy [9]) comprises a single round of mass treatment throughout an endemic focus, followed by surveys every 3 to 6 months to identify and treat individuals with suggestive skin lesions, plus their contacts. Recently, PCR on swabs of yaws-like, non-genital skin ulcers of children in Ghana and the Pacific Islands has revealed that 9%–47% contain DNA of *Haemophilus ducreyi*, the causative agent of chancroid, without detectable *T*. *pallidum* DNA [16,17], suggesting that PCR may be required for specific post-treatment surveillance. There is much more to learn about the epidemiology and pathogenesis of yaws and how best to use diagnostics to guide eradication.

## Will Susceptibility of Both Infections to Azithromycin Permit Mutually Beneficial Collaboration between Mass Treatment Programs for the Two Diseases?

Both the program to eradicate yaws and the program to eliminate trachoma recommend mass treatment (treatment of every resident member of a population)—the trachoma program once annually for up to 5 years (depending on baseline TF prevalence in 1- to 9-year-olds) before an impact survey, and the yaws program for one round only, followed by intermittent case finding (Table 1).

**Table 1:** Key characteristics of trachoma and yaws and their control.

	Trachoma	Yaws
Causative organism	*C*. *trachomatis* serovars A, B, Ba, and C	*T*. *pallidum* ssp. *pertenue*
Hypothesized routes of transmission	Mechanical transfer from infected eyes to uninfected eyes via fingers, fomites, and flies. No known animal reservoir.	Mechanical transfer from a primary or secondary yaws lesion to broken skin of an uninfected individual via direct contact or, possibly, flies. No known animal reservoir.
Clinical course	Repeated episodes of active (inflammatory) trachoma over many years cause conjunctival scarring, which in some individuals eventually draws the eyelashes inward so that they rub on and damage the cornea.	Multiple phases of disease. Primary yaws manifests as a papilloma at the inoculation site, which then ulcerates and scars over months. Untreated patients develop cutaneous, skeletal, and constitutional features of secondary yaws and, eventually, in some cases, tertiary yaws, characterized by necrotic and/or hypertrophic lesions of soft tissue and bone.
Epidemiology	Active trachoma is most common in pre-school-age children, with the prevalence of blinding consequences increasing with age; even in most hyper-intense transmission areas, trachomatous blindness is rare before adulthood.	Primary yaws is most common in school-age children. Secondary yaws occurs 1 to 24 months after untreated primary yaws. Tertiary yaws, now rare, occurs 5 or more years after untreated secondary yaws.
Sub-clinical infection provides a rationale for mass treatment of endemic populations	Yes. The proportion of individuals without clinical signs who have conjunctival *C*. *trachomatis* infection varies with the local intensity of transmission.	Yes. For each clinical case there may be more than five infected individuals without signs. Asymptomatic latent infection may persist for years between secondary and tertiary yaws.
Program goal	Elimination as a public health problem: reduction in the prevalence of trachomatous trichiasis (TT) unknown to the health system to <1 per 1,000 total population, and in the prevalence of TF in 1- to 9-year-olds to <5% in each district.	Eradication: no serologically positive children <5 years old and no new cases of active yaws for 3 consecutive years, in all countries where yaws has ever been known.
Strategy name	The “SAFE” strategy: surgery, antibiotics, facial cleanliness, and environmental improvement.	The WHO Morges strategy.
Recommended antibiotic	Azithromycin	Azithromycin
Recommended antibiotic dose	20 mg/kg, maximum 1 g	30 mg/kg, maximum 2 g
Recommended antibiotic schedule	Annual mass treatment for 3 or 5 years (depending on baseline prevalence) before re-survey.	One round of mass treatment, then targeted treatment (of all active clinical cases and their contacts) every 3 to 6 months.
Unit of implementation	District (the administrative unit for health care management, which, for purposes of clarification, consists of a population unit between 100,000 and 250,000 persons).	Conforming to the estimated extent of the endemic focus at baseline, and village- or community-based at follow-up and during surveillance.
Surveillance	Population-based surveys to estimate district-level prevalences of TT and TF, conducted two years after an impact assessment has shown that elimination goals have been reached.	Active surveillance in all villages using village volunteers and school teachers.

Apart from the number of rounds of mass treatment, there are three key differences in the way each program operationalises azithromycin use. First, for trachoma, the recommended dose is 20 mg/kg, to a maximum of 1 g. In yaws, the dose is 30 mg/kg, to a maximum of 2 g. However, for practical reasons, yaws programs determine dose by age, and trachoma programs determine dose by height, which probably makes the different dosage recommendations less programmatically significant than they might first appear. For example, in one setting, because trachoma’s height-based dosing algorithm is designed primarily to minimise under-dosing, the mean dose received by children aged 6 months to 9 years was >29 mg/kg [18]. Whether a lower dose might be effective for yaws, or a higher dose made routine in trachoma programs operating where yaws is co-endemic, are matters for further study and discussion. This should not prevent both programs from continuing work for public health benefit whilst developments are awaited.

Second, in trachoma programs, the recommended intervention unit is the district: a population of 100,000 to 250,000 people. For yaws, the recommended intervention unit changes with time, being flexible at baseline (conforming to the estimated extent of the endemic focus) and village- or community-based subsequently.

Third, trachoma elimination guidance stresses the importance of adjunctive measures alongside antibiotic distribution—namely, promotion of facial cleanliness and environmental improvement—to reduce transmission of ocular *C*. *trachomatis*. This multi-pronged approach implicitly recognizes the very low likelihood that infection will be cleared from a population through the use of one or several rounds of antibiotic treatment alone, and the consequent necessity to alter transmission intensity to maximise the impact of mass treatment. Though in cross-sectional data, access to hand-washing facilities is associated with lower risk of yaws infection [19], the WHO Morges strategy [9] does not explicitly incorporate hygiene-related interventions, but rather includes them under a general heading of “health promotion.” Regardless, yaws programs in some countries do recognize their potential importance [6].

## Where Do Trachoma and Yaws Both Occur?

**Fig 1:**
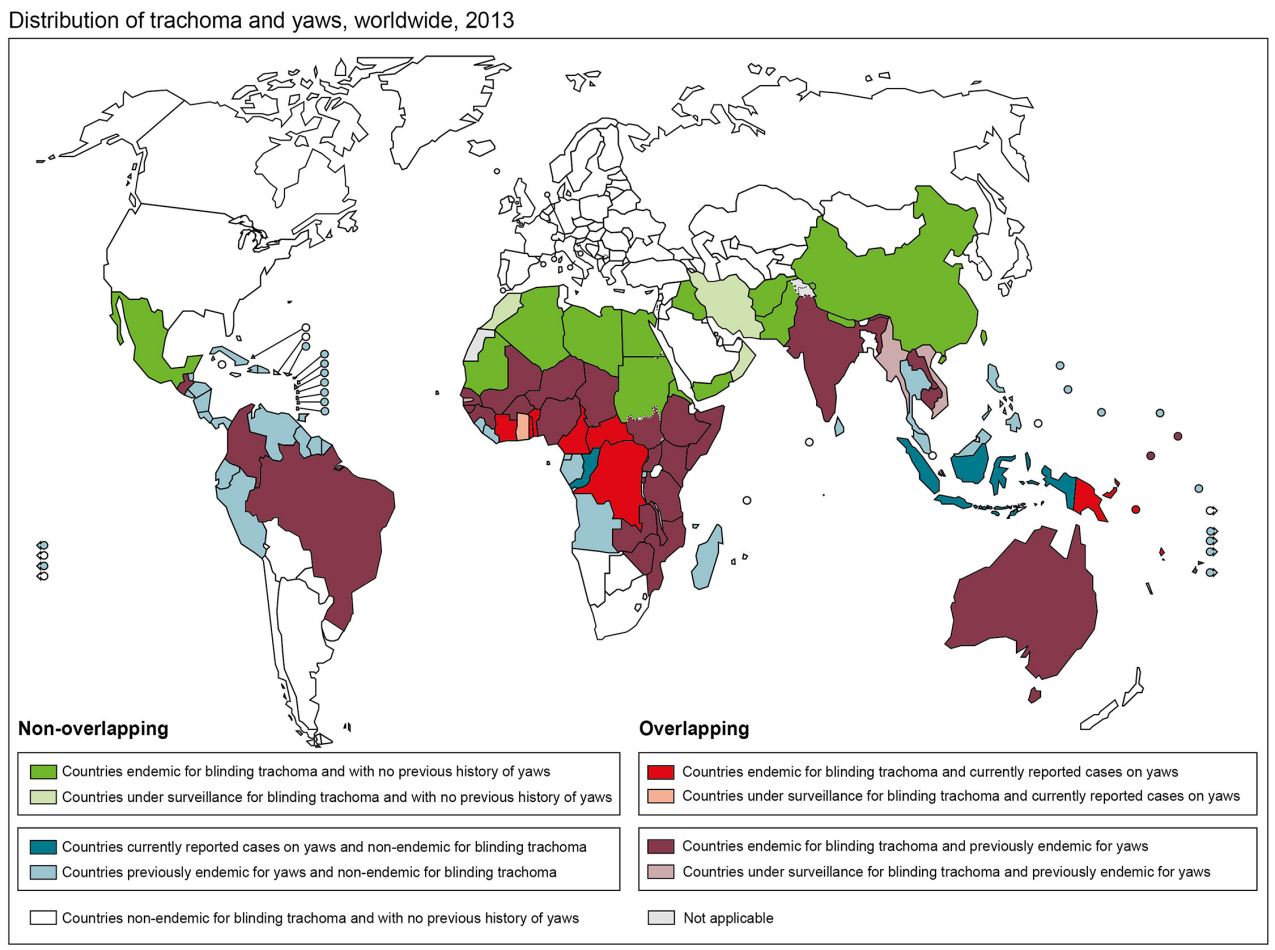
Categorization of endemicity of trachoma and yaws at country level, worldwide, 2013. For the purposes of this figure, a country is categorized as endemic for a disease based on its status in the Global Health Observatory database (http://www.who.int/gho/en/) as at 15 December 2014. A country is “endemic for blinding trachoma” in this database if it contains one or more administrative areas in which the most recent population-based prevalence data held by the Global Atlas of Trachoma (www.trachomaatlas.org) show the prevalence of TF in 1- to 9-year-olds to be ≥10% and/or the prevalence of TT in the whole population to be ≥0.1%. Papua New Guinea is additionally categorized as “endemic for blinding trachoma” on this map on the basis of preliminary survey work carried out by the Ministry of Health.

Resolving the complexities of different control strategies for these diseases will only be of practical relevance (to avoid treatment with doses of azithromycin that may be too low, and to avoid duplicate treatments by different programs) if there are populations endemic for both. The priority, therefore, is to delineate overlap. Trachoma is known or suspected to be of public health significance in 51 countries [20]. Building on two decades of less intensive data gathering, the Global Trachoma Mapping Project is currently working to establish the population-based prevalence of disease in each suspected endemic district for which accurate data are not available, as a prelude to implementation of elimination activities wherever required [21]; prevalence category data are posted to the open-access Global Atlas of Trachoma (www.trachomaatlas.org). The spatial distribution of yaws is much less certain. Clinical cases are now reported from 13 WHO member states, while the status of a further 73 previously endemic member states is unknown [22]. Work is currently underway to identify which previously yaws-endemic countries are most likely to still harbour cases of yaws and should be prioritized for urgent mapping, and which are likely to have achieved local interruption of transmission and should be targeted for formal certification. The geographic overlap of the two diseases at country level is shown in Fig 1.

Within endemic countries, the distribution of each disease is heterogeneous. Each “begins where the road ends,” in hot, tropical settings. But while trachoma is classically found in dry, dusty areas, yaws is restricted to humid environments, presumably due to the growth requirements of the organism.

**Fig 2:**
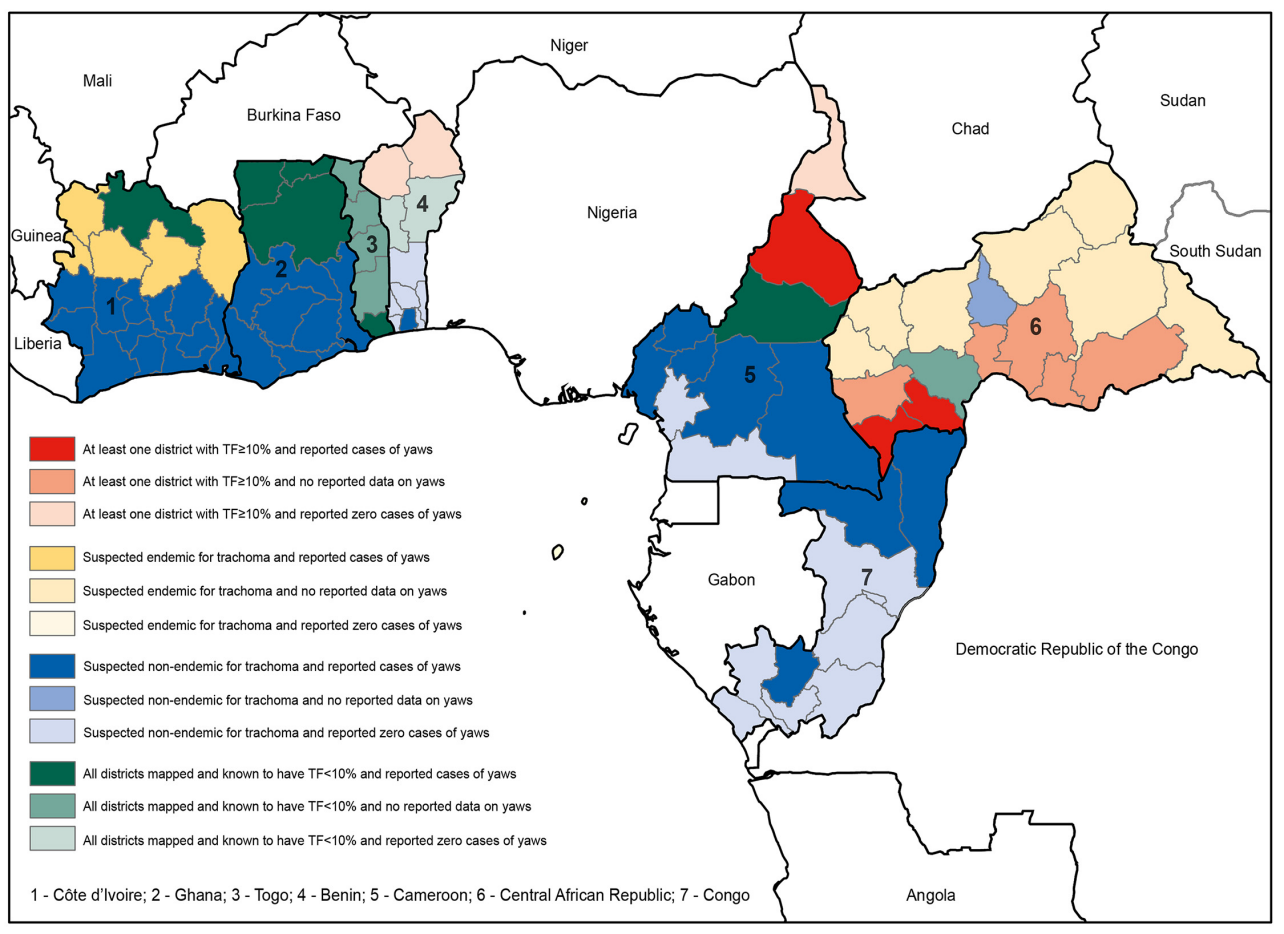
First-level administrative areas of countries in West and Central Africa co-endemic or potentially co-endemic for trachoma and yaws, based on reported clinical data on yaws in 2013 [23] and the most recent population-based prevalence data on TF held by the Global Atlas of Trachoma (www.trachomaatlas.org) as at 15 December 2014.

**Fig 3:**
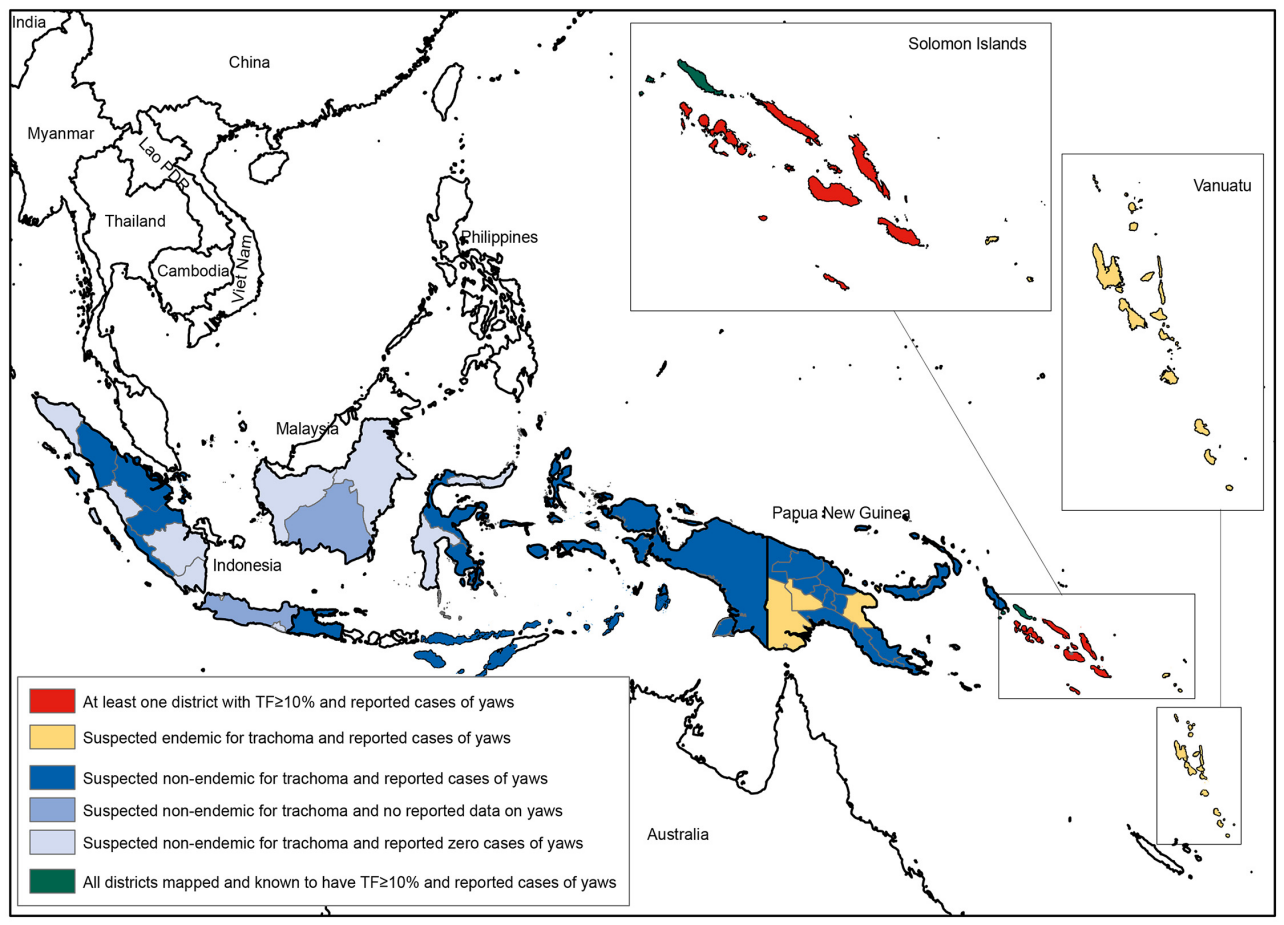
First-level administrative areas of countries in Southeast Asia and the Western Pacific co-endemic or potentially co-endemic for trachoma and yaws, based on reported clinical data on yaws in 2013 [23] and the most recent population-based prevalence data on TF held by the Global Atlas of Trachoma (www.trachomaatlas.org) as at 15 December 2014.

Available data on the spatial overlap of trachoma (www.trachomaatlas.org) and yaws [23] at the first sub-national administrative level are shown in Fig 2 for West Africa, and in Fig 3 for Southeast Asia and the Pacific. Table 2 shows the number and total population of districts known or suspected to be co-endemic for both diseases; it can be readily appreciated from these data that the area of potential overlap is far greater than the area for which overlap has been established. Much more effort is needed to delineate populations in which yaws is found.

**Table 2:** Active trachoma–yaws co-endemic and potentially co-endemic districts worldwide, 15 December 2014 (number of districts [estimated total population resident in those districts]). TF = trachomatous inflammation—follicular.

	Yaws cases currently reported	Yaws previously endemic
TF prevalence in 1- to 9-year-olds ≥10%	34 [4,928,234]	946 [141,298,660]
Trachoma suspected	27 [7,666,278]	226 [40,465,453]

Although the other endemic treponematoses, bejel and pinta, may also overlap geographically with trachoma and are likely to be responsive to azithromycin, even less is known about the distribution of these diseases than is known about that of yaws.

## Besides Mass Treatment with Azithromycin, Are There Other Potential Program Synergies?

Yes. First, as already mentioned, efforts are underway to map the prevalence of trachoma in every suspected endemic district. Where yaws is or may be co-endemic, it saves money and time to add collection of yaws data to trachoma mapping fieldwork, as has been done in the Solomon Islands [19], although the optimal evaluation unit size, sampling fraction, and sampling strategy are yet to be defined for yaws [24]. Regardless, the mapping infrastructure established for trachoma should be an asset for the yaws program.

Second, identification of individuals with TT is the first component of trachoma’s SAFE strategy. This involves screening large numbers of individuals for this potentially blinding condition, which is time-consuming but critical. Simultaneously screening for other endemic diseases, including yaws, is likely to maximise overall efficiencies for local health systems.

Third, the facial cleanliness and environmental improvement components of the SAFE strategy require participatory education in endemic communities, plus work to enhance the delivery of water and appropriate methods for disposal of human faeces. These measures are intended to reduce transmission of ocular *C*. *trachomatis*. Health education designed for trachoma control purposes could be expanded to provide a more comprehensive package, including, for example, encouraging appropriate ulcer care to limit transmission of *T*. *pallidum* ssp. *pertenue*. Similarly, improvement in water supplies is likely to also benefit yaws control: good personal hygiene contributes to reducing infection spread.

Fourth, in the impact assessment and validation of elimination or certification of eradication stages of each program, in part because of the difficulty in making definitive diagnoses on clinical grounds, it is possible that serological and/or nucleic acid amplification-based diagnostics will be required (though introducing these things for trachoma would require more evidence and a change to current guidance [25]). Whilst detection of nucleic acid from the pathogens of yaws and trachoma may not be perfectly suited to an integrated diagnostic system, in part because the samples (conjunctival swab, ulcer swab) are different, multiplex testing in the serology laboratory can provide data on the presence or absence of antibodies against multiple antigens from a sample of <1 μL of blood, facilitating use in integrated surveillance of various tropical and waterborne diseases [26]. Antibody-based epidemiological assessment for yaws and other treponemal diseases has been in place for more than five decades [19,27]. The concept is presently being evaluated for trachoma, but early work has demonstrated promise, indicating the potential utility of seroprevalence in young children as an indicator of altered transmission dynamics in endemic populations [28]. There may ultimately be scope for collaboration here, following on from previous successful collaboration in the research setting [19].

## Conclusions

The global yaws and trachoma programs have much to learn from each other’s histories, current projects, and future plans. Where the two diseases are co-endemic, considerable benefits may be accrued by each program by exploiting opportunities to work together toward two important public health goals.

**Key Learning Points**

1. Both yaws and trachoma require interventions delivered to the community, which may include one or more rounds of mass treatment with azithromycin.
2. The goals of the yaws and trachoma programs are different, and therefore the implementation of interventions may not be fully aligned. In addition, the recommended doses of azithromycin, the nature of the intervention unit, and the application of adjunctive, non-antibiotic interventions are all currently different.
3. Much is known about the current geographical distribution of trachoma. Much less is known about the current geographical distribution of yaws. Further work to delineate the epidemiology of yaws would be helpful for exploring to what extent potential synergies between the two programs should be pursued.
4. In addition to potential synergies relating to mass treatment with azithomycin, the two programs might each be able to benefit through collaborations on mapping, case finding, health education, environmental improvement, and surveillance.
